# Increasing gene dosage greatly enhances recombinant expression of aquaporins in *Pichia pastoris*

**DOI:** 10.1186/1472-6750-11-47

**Published:** 2011-05-10

**Authors:** Kristina Nordén, Maria Agemark, Jonas ÅH Danielson, Erik Alexandersson, Per Kjellbom, Urban Johanson

**Affiliations:** 1 Department of Biochemistry and Structural Biology, Center for Molecular Protein Science, Center for Chemistry and Chemical Engineering, Lund University, PO Box 124, S-221 00 Lund, Sweden; 2 Institute for Wine Biotechnology, Faculty of AgriSciences Stellenbosch University, Private Bag X1, 7602 Matieland, South Africa

**Keywords:** *Pichia pastoris*, aquaporins, Major Intrinsic Proteins, qPCR

## Abstract

**Background:**

When performing functional and structural studies, large quantities of pure protein are desired. Most membrane proteins are however not abundantly expressed in their native tissues, which in general rules out purification from natural sources. Heterologous expression, especially of eukaryotic membrane proteins, has also proven to be challenging. The development of expression systems in insect cells and yeasts has resulted in an increase in successful overexpression of eukaryotic proteins. High yields of membrane protein from such hosts are however not guaranteed and several, to a large extent unexplored, factors may influence recombinant expression levels. In this report we have used four isoforms of aquaporins to systematically investigate parameters that may affect protein yield when overexpressing membrane proteins in the yeast *Pichia pastoris*.

**Results:**

By comparing clones carrying a single gene copy, we show a remarkable variation in recombinant protein expression between isoforms and that the poor expression observed for one of the isoforms could only in part be explained by reduced transcript levels. Furthermore, we show that heterologous expression levels of all four aquaporin isoforms strongly respond to an increase in recombinant gene dosage, independent of the amount of protein expressed from a single gene copy. We also demonstrate that the increased expression does not appear to compromise the protein folding and the membrane localisation.

**Conclusions:**

We report a convenient and robust method based on qPCR to determine recombinant gene dosage. The method is generic for all constructs based on the pPICZ vectors and offers an inexpensive, quick and reliable means of characterising recombinant *P. pastoris *clones. By using this method we show that: (1) heterologous expression of all aquaporins investigated respond strongly to an increase in recombinant gene dosage (2) expression from a single recombinant gene copy varies in an isoform dependent manner (3) the poor expression observed for AtSIP1;1 is mainly caused by posttranscriptional limitations. The protein folding and membrane localisation seems to be unaffected by increased expression levels. Thus a screen for elevated gene dosage can routinely be performed for identification of *P. pastoris *clones with high expression levels of aquaporins and other classes of membrane proteins.

## Background

Most membrane proteins are not abundantly expressed in their native tissues making it hard to recover enough material for functional and structural studies. In addition several isoforms with similar biochemical properties are often co-expressed which further complicates the purification process. Hence, efficient heterologous systems for overexpression are important for biochemical characterisation of membrane proteins, which is also reflected by the increase in membrane protein structures derived from heterologously expressed material over the last decade [1,2]. *Escherichia coli *is still the most common host for membrane protein overexpression but is often not efficient when expressing eukaryotic membrane proteins [3]. Yeasts have emerged as alternative hosts for expression, having the benefits of being inexpensive and easy to manipulate in addition to having the ability to grow to high cell densities [4]. Furthermore, they possess the eukaryotic machinery for post translational modifications [5] and the eukaryotic translocon complex [6] that might be required for synthesis of a correctly folded and fully functional membrane protein.

The use of the methylotrophic yeast *Pichia pastoris *for heterologous protein production was developed in the 1980s [7] and it has proven to be a successful host when overexpressing soluble proteins [8] as well as several different classes of membrane proteins, including a peptide transporter [9], ion channels [10,11], ion pumps [12,13], G-protein coupled receptors [3,14-16], ABC transporters [17] and glucose transporters [18,19]. Due to its ability to grow to high cell densities and to its exceptionally strong and inducible *AOX1 *promoter [8], *P. pastoris *has become a potent alternative to the more studied baker's yeast *Saccharomyces cerevisiae *as a eukaryotic tool for recombinant protein production.

A number of factors can potentially affect the outcome of heterologous protein expression in *P. pastoris*, including the properties of the nucleotide sequence (e.g. codon composition, AT-content and secondary structure), mode of sequence insertion into the genome, choice of host strain, mode of expression, methanol utilisation phenotype and cultivation conditions [8,20]. It has however been stated that when expressing protein from a single recombinant gene copy, the transcript level is most likely to be limiting in the protein production. It is therefore suggested that insertion of multiple recombinant gene copies should be aimed for in order to obtain maximal protein yields [21]. When using a *P. pastoris *expression vector carrying the *Sh ble *gene which encodes a protein conferring resistance towards the antibiotic zeocin, a selection on varying antibiotic concentrations can be applied, as described in the Invitrogen Easy Select™ *Pichia *expression kit manual [22]. The level of antibiotic resistance will reflect the number of plasmids that have been incorporated into the *P. pastoris *genome, that is, the recombinant gene dosage. Numerous studies have shown that increased number of inserted sequences can indeed boost the heterologous expression of soluble proteins [23-32]. Concerning membrane proteins, the correlation between increased recombinant gene dosage and protein expression is less well studied and the results obtained are contradictory. There is one report where increased gene dosage, as estimated by semi quantitative DNA dot blotting, rendered significantly increased recombinant expression of a Na^+^, K^+ ^-ATPase [12]. In two other expression studies on GPCR:s, limited [15] or no [33] effect of increasing recombinant gene dosage was seen, suggesting that other factors were limiting the steady state expression levels.

Another class of membrane proteins that, in addition to the ones previously mentioned, has been successfully overexpressed in *P. pastoris *is the Membrane Intrinsic Proteins (MIPs), also called aquaporins [34-38]. Members of this superfamily of integral membrane proteins are present in nearly all living organisms [39]. Aquaporins are proteins with a number of functions, whereof the most significant one is the facilitation of water transport over the membranes of the cell and the maintenance of water homeostasis in the organism [40]. Functional and structural studies have led to a broadening of the knowledge base around these proteins [41]. However, since many questions regarding the aquaporin function and structure remain unanswered and more studies are needed to complete the picture, convenient methods for routinely obtaining high expression levels of recombinant aquaporins in *P. pastoris *are desirable. Moreover, since the aquaporins are integral, they can serve as model proteins for other membrane spanning proteins and methods developed based on recombinant aquaporin expression might hence be applicable also in a broader context.

In this study, we systematically investigate how recombinant gene dosage influences expression levels of both mammalian (HsAQP5 and HsAQP8) and plant (SoPIP1;2 and AtSIP1;1) aquaporins in *P. pastoris*. We show that increased copy number leads to elevated heterologous expression of all the four members of the aquaporin family studied. Interestingly this includes AtSIP1;1, for which expression appears to be restricted due to impaired transcription and translation. A screen for multiple copy integrants should hence be a part of the routine optimisation strategy when overexpressing aquaporins in this host. We also present a method for fast, cheap and reliable determination of integrated plasmid copy number using qPCR. This method makes it possible to easily characterise the transformants and to correlate expression levels with gene dosage which is valuable when trying to find the *P. pastoris *clones with optimal characteristics for heterologous protein expression.

## Results

### Expression analysis

**Figure 1:**
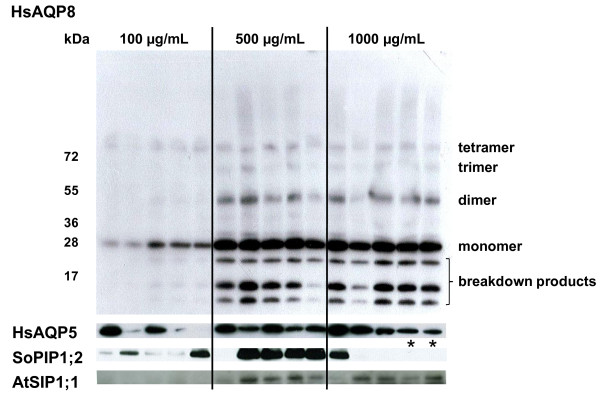
**Correlation between heterologous expression and zeocin resistance for the four different aquaporins**. Western blot with crude cell extract from five representative clones from each zeocin selection level (100, 500 or 1000 μg zeocin/mL) and each aquaporin isoform (HsAQP8, HsAQP5, SoPIP1;2 and AtSIP1;1). HsAQP8 samples (top) show the characteristic aquaporin pattern with monomer, dimer, trimer and tetramer and also indicate that degradation seems to occur. For simplicity only the major band, corresponding to the monomer, is shown for the other aquaporins. (Asterisks indicate clones carrying the SoPIP1;2 construct recovered from selection at 2000 μg zeocin/mL). There is a correlation between recombinant aquaporin expression and level of antibiotic resistance.

Since the level of resistance towards the antibiotic zeocin is supposed to reflect the number of inserted vector sequences in *P. pastoris*, and potentially also the level of expression, clones carrying recombinant DNA encoding each of the four aquaporin isoforms were selected at varying zeocin concentrations. For each isoform, five clones (or less if as many as five could not be recovered, see methods) at each antibiotic resistance level were induced in small scale and crude cell extracts were analysed by immunoblotting using an antibody directed towards the C-terminal His-tag of the recombinant proteins. Western blots from the small scale expression show a clear difference in expression levels among the different clones (Figure 1). Although, there are large differences in expression levels between the different aquaporin family members (see below) it is apparent that, for each individual isoform, the yeast cells selected on zeocin concentrations higher than 100 μg/mL generally express larger amounts of heterologous protein. One exception, however, are the SoPIP1;2-clones selected at 1000 or 2000 μg/mL zeocin which were obtained only after an extended incubation of the selection plates.

**Figure 2:**
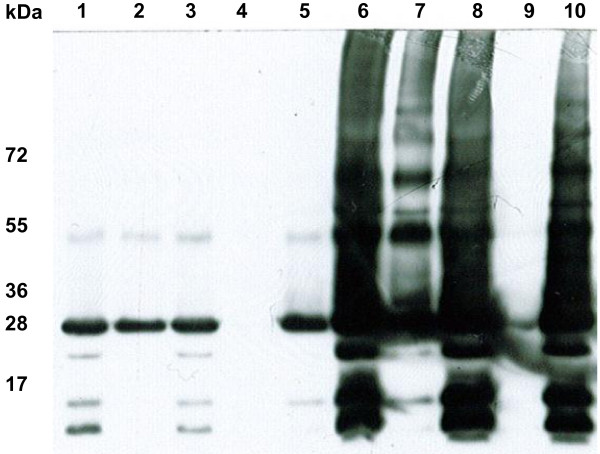
**Protein folding and membrane localisation**. Fractions obtained during the large scale membrane preparation from *P. pastoris *clones expressing moderate (HsAQP8:**100**:2) or high (HsAQP8:**1000**:1) levels of recombinant HsAQP8 analysed by immunoblotting. Lane 1-5: HsAQP8:**100**:2 fractions (crude cell extract (1), 1400 *g *pellet (2), 1400 *g *supernatant (3), 150 000 *g *supernatant (4) and membrane fraction (5)). Lane 6-10: HsAQP8:**1000**:1 fractions (crude cell extract (6), 1400 *g *pellet (7), 1400 *g *supernatant (8), 150 000 *g *supernatant (9), membrane fraction (10)). All fractions recovered were, prior to SDS-PAGE, diluted to a normalised volume corresponding to 270 mL/fraction whereof the same volume for each fraction was loaded on the gel. The protein folding and membrane localisation do not seem to be compromised by increased protein expression.

To verify that the elevated aquaporin expression of the clones with high zeocin resistance levels did not compromise the protein folding and the membrane insertion, a large scale expression test and subsequent membrane preparation was made of a clone expressing high levels of HsAQP8 (HsAQP8:**1000**:1) with a clone with modest expression (HsAQP8:**100**:2) as a reference applying exactly the same protocol for both clones. To be able to directly compare the HsAQP8 content in the fractions derived from the HsAQP8:**1000**:1 and the HsAQP8:**100**:2 clones respectively, each fraction was diluted to a normalized volume prior to analysis with western blots. Immunoblotting (Figure 2) showed that some protein was retained in the cell debris pellet (lane 2 and 7 respectively), but that the majority of the protein was situated in the purified membrane (lane 5 and 10 respectively). Also at high expression levels, the protein formed the multimeric complexes characteristic of correctly folded aquaporins. For the HsAQP8:**1000**:1 clone, some faint but distinct bands also appeared in-between the bands corresponding to the AQP8 multimers and these could correspond to AQP8 protein subjected to post translational modifications. These were not seen when analyzing the fractions from the HsAQP8:**100**:2 clone, since significantly less AQP8 protein was present in these fractions.

### Analysis of copy number by qPCR

To be able to correlate the recombinant protein expression level with the number of inserted gene copies a method based on qPCR was developed. The method takes advantage of the fact that the *AOX1 *TT region, that is part of the pPICZB plasmid, is incorporated in the *P. pastoris *genome together with the gene to be expressed. Quantification of the *AOX1 *TT region will hence give an indirect measurement of the recombinant gene dosage, independent of which protein is to be overexpressed.

**Figure 3:**
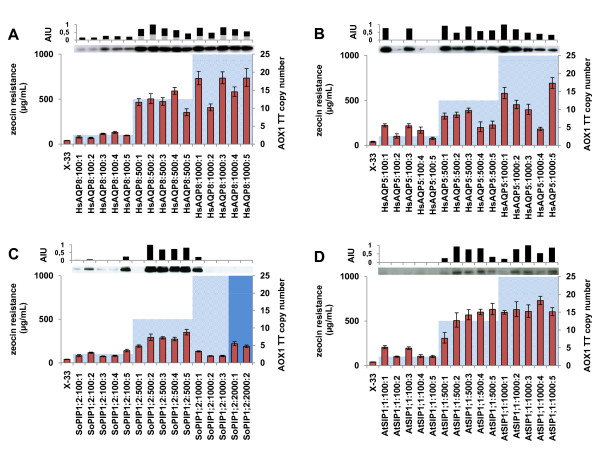
**Correlation between the recombinant gene dosage and the expression levels of the aquaporins**. Diagrams showing the μg/mL zeocin selection level and the *AOX1 *TT copy numbers ± SD of clones carrying the HsAQP8 (A), HsAQP5 (B), SoPIP1;2 (C) and AtSIP1;1 (D) constructs. For the SoPIP1;2 construct, two clones were recovered from the 2000 μg/mL zeocin selection. This higher antibiotic resistance is indicated by a darker colour in the diagram. The *AOX1 *TT copy numbers include the native *AOX1 *TT sequence and the sequences derived from the incorporated plasmids. For the X-33 wild type strain, included as a standard, where no vector derived *AOX1 *TT sequences are present, a 1:1 ratio between the *AOX1 *TT sequence and the internal reference sequence is expected and a value of 1 ± SD is calculated. By subtracting this value from the *AOX1 *TT copy numbers obtained for the recombinant clones, the aquaporin gene copy numbers were subsequently calculated (see methods and Figure 6). Included are also western blots showing recombinant expression levels and diagrams summarising the densitometric quantification of each of the lanes with total protein (black bars) and degradation products (grey bars) indicated. Values are given as Arbitrary Intensity Units (AIU:s). There is a correlation between aquaporin gene copy number and antibiotic selection level and between aquaporin expression and aquaporin gene copy number.

The qPCR data shows, as expected, that the aquaporin gene copy number increases with elevated resistance towards zeocin (Figure 3). Generally, clones selected on 100 μg/mL zeocin tend to harbour 1-5 copies of the inserted plasmid, selection on 500 μg/mL renders clones with 4-15 plasmid copies and selection on 1000 μg/mL could give rise to clones with as many as 17 incorporated heterologous DNA sequences. The analysis also shows that low copy SoPIP1;2 clones were recovered at high antibiotic concentrations indicating that the zeocin selection method used did not completely exclude the occurrence of so called false positives (see discussion). When comparing the qPCR data with the expression levels of the different clones it is obvious that there is a correlation between gene dosage and recombinant protein expression level. The linear regression correlation coefficients (r^2^) for HsAQP5, HsAQP8, SoPIP1;2 and AtSIP1;1 are 0.21, 0.72, 0.70 and 0.62, respectively. The low r^2 ^value for HsAQP5 is due to the low expression of the high copy clone HsAQP5:**1000**:5 and without this data point the r^2 ^value is 0.52.

### Comparison of single copy aquaporin clones

**Figure 4:**
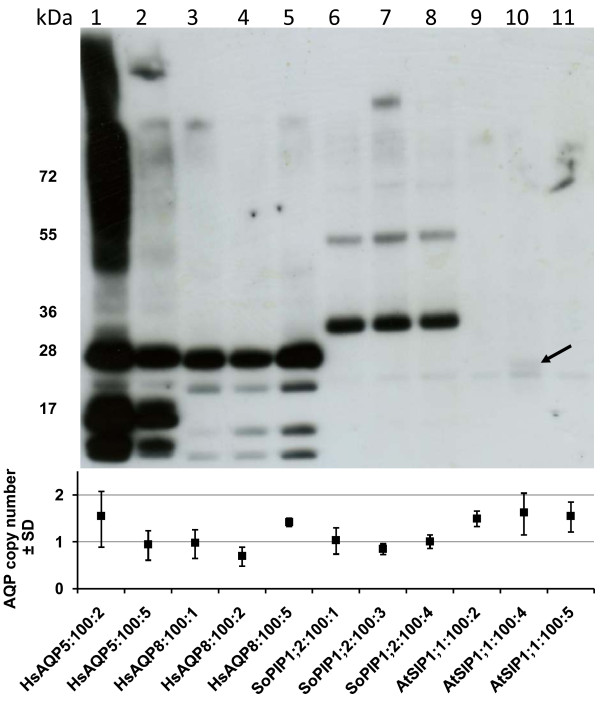
**Recombinant expression levels of aquaporin isoforms in single copy clones**. Western blot with crude cell extracts from single copy representatives of the four different aquaporins (top). The calculated aquaporin copy number ± SD of each clone is also included (bottom). The AtSIP1;1 isoform is poorly expressed as compared with the other isoforms and the very faint monomeric band for one of the clones is indicated by an arrow.

In order to compare expression levels of different aquaporin isoforms, crude cell extracts from induced, potentially single copy, aquaporin clones were analysed on the same western blot (Figure 4). The blot shows similar expression levels of HsAQP5, HsAQP8 and SoPIP1;2, with the two human isoforms giving a slightly stronger signal. It is noticeable that one HsAQP5- and one HsAQP8-clone that had been designated copy numbers around 1.5 (lane 1 and 5) are more likely to carry two copies of the respective genes, since they show significantly higher expression than the clones with copy numbers closer to one. The most prominent feature though is the poor expression of AtSIP1;1, which monomer (indicated by an arrow) is on the verge of detection.

### Quantification of AtSIP1;1 mRNA and modification of the consensus start sequence

**Figure 5:**
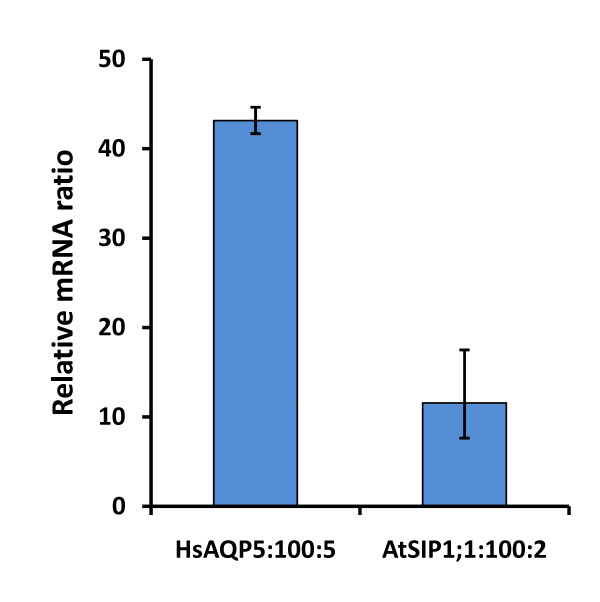
**AtSIP1;1 and HsAQP5 transcript levels**. mRNA levels of induced single copy HsAQP5- (HsAQP5:**100**:5) and AtSIP1;1- (AtSIP1;1:**100**:2) clones. Triplicate cultures were analysed by qPCR. Values are given as the average relative ratio between aquaporin mRNA and actin mRNA. AtSIP1;1 mRNA levels are low as compared with the AQP5 transcript levels but still substantially higher than these of the reference (actin).

To investigate whether the poor AtSIP1;1 expression was caused by low transcript levels an mRNA quantification experiment using qPCR was performed. The results, summarised in Figure 5, show that upon induction the single copy AtSIP1;1 clone (AtSIP1;1:**100**:2) produces about 4 times less recombinant mRNA as compared with the single copy HsAQP5 clone (HsAQP5:**100**:5). However, the AtSIP1;1 transcript level is still more than 11 fold higher than that of actin, that was used as a reference.

Since the AtSIP1;1 construct was cloned with restriction enzymes different from those used for the other three aquaporins, it differed from the consensus start sequence [22] in one nucleotide (CAAAATGTCT → GAAAATGTCT). To investigate whether this deviation was causing the low AtSIP1;1 expression levels, a new construct carrying the consensus start sequence was made and protein expression of a clone selected on 100 μg/ml was compared with that of the AtSIP1;1:**100**:5 clone. Expression increased slightly but was still poor as compared with that of the single copy HsAQP5 clone (HsAQP5:**100**:5) that was included as a reference (data not shown).

## Discussion

The methylotrophic yeast *P. pastoris *has, during the last decades, increased in popularity as a eukaryotic host for recombinant protein expression [7]. Numerous reports have described successful overexpression of soluble proteins as well as of membrane proteins in this system, but sufficient protein yields have not always been obtained. Several methods have been described that could be used to improve the outcome of expression trials, among which an optimisation of recombinant gene dosage has proven to be one of the most potent ones [21]. When it comes to the aquaporin family of membrane proteins, optimisation of the nucleotide sequence both regarding codon composition and AT content [38] and controlled growth in bioreactors [36] have been reported to improve the yields of heterologous protein. However, in no reports has a systematic examination of the effect of gene dosage on recombinant aquaporin expression in *P. pastoris *been done and results obtained for other membrane proteins [12,15,33] are based on less precise DNA quantification methods. Neither has transcript levels for different genes with the same promoter and copy number been compared. A better understanding of how these factors may affect the yield will facilitate the design of future membrane protein expression experiments.

As for many other proteins expressed in *P. pastoris *[23,29,30,32], recombinant aquaporin expression was shown to be intimately connected to the level of resistance towards the antibiotic. In general clones selected at 500 or 1000 μg zeocin/mL are more likely to express high levels of recombinant protein as compared with the ones selected at 100 μg/mL. In this study we chose to perform a two step antibiotic selection in order to take full advantage of all the clones generated in each transformation. By doing a first selection at 100 μg zeocin/mL all recombinant clones were recovered and these were then subjected to higher zeocin concentrations. The benefit of this method is that the risk of losing potential high expressing clones by subjecting them to lethal levels of antibiotic is eliminated. The drawback is that several clones from the same origin may be recovered during the second selection event, as possibly seen for the SoPIP1;2 clones (discussed below).

The large scale membrane preparation from a clone expressing high levels of one of the aquaporins (HsAQP8) showed that the folding and the targeting to the membrane seem to be unaffected by the increased work load that is put on the protein production and the membrane insertion machinery. Comparison with a clone with low expression level showed that the majority of the protein ends up in the membrane fraction in both cases and, most importantly, that the membrane fraction from the highly expressing clone contains substantially larger amounts of recombinant protein (Figure 2). Some degradation seems to occur and this could probably be avoided by optimisation of the cell breakage procedure or by expression in a protease deficient strain [8]. When combining the high amount of correctly folded and targeted protein in multi-copy transformants with the high cell densities obtained in bioreactor cultures, exceptional membrane protein yields could be achieved. This was seen for one multi-copy HsAQP5 clone with high levels of recombinant protein expression, where 145 mg of functional protein was purified from 1 L fermenter culture (unpublished data). Protein from this clone was also used for crystallisation and rendered well ordered crystals diffracting to 3.1 Å [34,42], confirming the high quality of the expressed protein.

When copy number was determined it was confirmed that the variation in antibiotic tolerance was due to differences in recombinant gene dosage among the different clones. Assuming that the *Sh ble *gene is incorporated at the same ratio as the *AOX1 *TT sequence it generally seems like the presence of 1 copy of the *Sh ble *zeocin resistance gene is the minimum requirement for growth at 100 μg zeocin/mL, 4 copies at 500 μg/mL and 9 copies at 1000 μg/mL, but clones with as many as 17 gene copies were recovered from plates with the highest antibiotic concentration. The results obtained for the aquaporin isoforms included in this study suggest a trend between elevated gene dosage and increased recombinant expression, also for SoPIP1;2 where, even though only intermediate copy numbers were observed, expression increased by a factor of 70. This is in accordance with the results obtained when expressing a Na^+^K^+^-ATPase [12], but in contradiction with results from a GPCR expression trial [33]. Thus it seems like the multi copy strategy may not be the universal solution to all problems encountered when trying to over express MP:s, since there may be several factors limiting the recombinant protein production. One such bottleneck might be a limitation of the membranes to accommodate the expressed protein, resulting in a maximal upper limit for protein expression when gene dosage is further increased. Indeed, in our study we observe a plateau in the average expression when increasing gene copy number as exemplified by HsAQP5. The average expression of the 500- and 1000-selected clones were determined to 0.69 and 0.58 arbitrary units, respectively when the corresponding average gene dosage increased from 6.4 and 10.5 respectively. Still, a 147 fold increase in HsAQP5 expression level was obtained utilising the multi copy strategy. This demonstrates the potential of this method.

It is remarkable that from the 100 μg zeocin/mL selection, at least 9 out of 20 clones (45%) proved to harbour more than one recombinant gene copy. This frequency of multiple gene insertion events is notably higher than the expected 1-10% suggested in the EasySelect™ *Pichia *Expression Kit manual (Invitrogen). Possibly this high multi-copy frequency could have arisen during the two step zeocin selection due to posttransformational vector amplification which has been reported to occur when subjecting recombinant *P. pastoris *clones to gradually increasing concentrations of antibiotic [43]. In that report posttransformational vector amplification upon recurring antibiotic selection lead to elevated gene dosage in 40% of the clones studied. That frequency is comparable with the 45% seen in this study.

One problem that is commonly encountered when expressing protein in *P. pastoris *is the occurrence of false positives, that is, clones that have the capacity to grow at high antibiotic levels but express little or no recombinant protein [8]. False positives were also observed in this study. For the clones carrying the SoPIP1;2 construct, five out of the ten clones selected at the three highest zeocin levels (500 μg/mL, 1000 μg/mL and 2000 μg/mL) showed little or no recombinant protein expression. qPCR results showed that two of these clones (SoPIP1;2:**1000**:2 and SoPIP1;2:**1000**:3) carried only one copy of the *SoPIP1;2 *gene and three of them (SoPIP1;2:**500**:1, SoPIP1;2:**2000**:1 and SoPIP1;2:**2000**:2) were assigned copy numbers close to four. Probably the three latter false positives have a common origin in a clone with faulty heterologous expression. Since all SoPIP1;2 clones selected at 1000 or 2000 μg zeocin/mL were shown to harbour unexpectedly low numbers of integrated plasmid, the antibiotic selection for recovering multiple insertions seems to have failed. The only factor that differed in this selection as compared with the ones done for the other three constructs was the incubation time, which was increased from three to four days. Possibly there was degradation of zeocin over time or this procedure favoured the selection of mutations that increased resistance in alternative ways, not coupled to gene copy number. Regardless of the underlying mechanism it is apparent that extended incubation should be avoided in order to exclude the formation of false positives.

The results from the SoPIP1;2 selection emphasise the importance of evaluating the outcome of the antibiotic selection not only at the protein level, but also at the DNA level, to ensure that the selection is efficient and that satisfactory gene dosage is obtained. If, as in the case with SoPIP1;2, gene dosage turns out to be moderate one should consider redoing the transformation and selection in order to obtain clones with maximal gene dosage since they may be superior protein producers. The qPCR based method presented here offers a convenient and reliable means of characterizing recombinant *P. pastoris *clones and could be included in the standard protocols when screening for multi-copy transformants. The big advantage of this new method as compared with other qPCR based methods used to verify recombinant gene dosage in *P. pastoris *[32,44] is its versatility. Since the primers used are designed to amplify a part of the vector sequence rather than the specific gene, it can be used to quantify any recombinant gene sequence sitting in an expression vector carrying the *AOX1 *TT sequence.

One of the aquaporins, AtSIP1;1, proved to express very low levels of recombinant protein as compared with the other aquaporin isoforms. Analysis of the AtSIP1;1 mRNA levels showed that AtSIP1;1 transcripts were not as abundant as HsAQP5 transcripts. Due to the presence of an *Eco*RI cleavage site within the AtSIP1;1 coding sequence, the *Eco*RI restriction enzyme could not, as for the other three aquaporin isoforms, be used when creating the AtSIP1;1 construct. It is possible that the deviating site of insertion into the expression vector had an impact on the mRNA levels. However, AtSIP1;1 mRNA levels were still considerably higher than these of actin. Recombinant protein expression could hence be expected to be lower than that of HsAQP5 but should still be readily detectable with a western blot, unless there are obstructions in the translation that could contribute to the poor AtSIP1;1 expression. To exclude the possible adverse effects on translation of a deviation in the nucleotide sequence proximate to the start codon, a new construct carrying the correct *P. pastoris *consensus start sequence was made. This modification did not lead to significant improvement of the recombinant protein expression, and hence that factor was excluded as being a major obstacle to efficient recombinant AtSIP1;1 expression.

There are no other obvious features at the nucleotide level (such as high AT content or poor codon adaptation) that distinguish AtSIP1;1 from the other three aquaporin cDNA sequences studied. However, the AtSIP1;1 protein is the most divergent isoform (Table 1) and the only one of the four aquaporins that, natively, has been reported to reside in the membrane of the endoplasmic reticulum (ER) [45], whilst HsAQP5, HsAQP8 and SoPIP1;2 have been shown to be localised to the plasma membrane [39,46]. It is possible that the AtSIP1;1 is also retained in the ER in *P. pastoris *and that the accumulation of recombinant protein in these membranes limits the yield. Whatever the reason for the poor AtSIP1;1 expression, the fact that it could, to some extent, be overcome by elevating the gene dosage is encouraging and emphasises the potency of the multi-copy selection procedure.

**Table 1:** Pairwise identity and similarity between aquaporin isoforms

	HsAQP5	HsAQP8	SoPIP1;2	AtSIP1;1
**HsAQP5**	-	46.0	42.8	35.8
**HsAQP8**	30.0	-	39.2	35.4
**SoPIP1;2**	27.4	27.4	-	33.1
**AtSIP1;1**	20.9	22.3	20.7	-

Pairwise identities (below diagonal) and similarities (above the diagonal) were calculated using the program Needle at the EBI server http://srs.ebi.ac.uk

## Conclusions

In this study we present a method based on qPCR to determine recombinant gene copy number, that facilitates the validation of the antibiotic selection and the characterisation of recombinant *P. pastoris *clones. Employing this method we have systematically investigated factors that could influence recombinant aquaporin expression in *P. pastoris*. We conclude that expression of correctly folded and targeted aquaporins is strongly dependent on recombinant gene dosage and also that increasing the gene dosage could, to some extent, help improving poor expression caused by inefficiency at the transcriptional and translational levels.

## Methods

### Selection of multi-copy P. pastoris transformants and expression analysis

#### Cloning

The cDNA of the different aquaporin isoforms (*HsAQP5, HsAQP8, SoPIP1;2 *and *AtSIP1;1*) was amplified with the primers listed in Additional file 1, Table S1. The respective PCR products were cloned into the pPICZB vector (Invitrogen) between the *Eco*RI and *Not*I sites (*HsAQP5, HsAQP8 *and *SoPIP1;2*) or the *Xho*I and *Not*I sites (*AtSIP1;1*) conferring the addition of a myc antibody epitope and His_6_-tag to the C-terminus of the amino acid sequence. The resulting plasmids were sequenced in order to confirm the correct nucleotide sequence.

The four constructs were linearised by *Sac*I and complete linearisation was confirmed by agarose gel electrophoresis. 10 μg of linearised plasmid was transformed into competent wild type X-33 *P. pastoris *cells by electroporation according to the EasySelect™ *Pichia *Expression Kit Manual (Invitrogen).

#### Zeocin selection

Transformants were initially selected on YPD (1% w/v yeast extract, 2% w/v peptone, 2% w/v dextrose) agar plates containing 100 μg/mL Zeocin. After 3 days, transformants were resuspended in YPD medium that was added directly to the plates. Cells from the same transformation event were pooled and plated onto YPD agar plates containing 100, 500, 1000 or 2000 μg/mL Zeocin to select for clones with higher resistance level. Colonies that appeared within 3 days after the second plating event were believed to be true positives and 5 representatives from each construct and zeocin level were further analysed. For the HsAQP5, HsAQP8 and AtSIP1;1-constructs, the selection at 2000 μg zeocin/mL rendered no colonies. For the SoPIP1;2 construct no colonies had appeared on the plates containing the two highest antibiotic concentrations after three days, hence these were left to incubate for another 24 h where after colonies appeared and 3 clones from the 1000 μg/ml and 2 clones from the 2000 μg/mL-plates were further analysed. The selected clones were assigned clone IDs describing the isoform, the antibiotic selection level in μg/mL (bold) and the number of the clone (e. g. HsAQP5:**100**:1).

#### Small scale expression screen

To analyse the protein expression levels of the selected clones, a small scale protein expression screen was performed. To generate biomass, transformants were grown in 5 mL BMGY (1% w/v yeast extract, 2% w/v peptone, 100 mM potassium phosphate pH 6.0, 1.34% w/v yeast nitrogen base, 4 × 10^-5^% w/v biotin, 1% v/v glycerol) over night. Cells were harvested and resuspended in 5 mL BMMY (1% w/v yeast extract, 2% w/v peptone, 100 mM potassium phosphate pH 6.0, 1.34% w/v yeast nitrogen base, 4 × 10^-5^% w/v biotin, 0.5% v/v methanol) to an optical density at 600 nm (OD_600_) = 1. Induction was maintained for 72 h by addition of methanol to a final concentration of 0.5% v/v every 24 h. 3 h after the last methanol addition 20 OD_600 _units of cells from each clone were harvested by centrifugation and resuspended in 100 μL breaking buffer (50 mM NaPO_4 _pH 7.4, 1 mM EDTA, 5% v/v glycerol, 1 mM PMSF). Glass beads were added and cells were broken by vortexing 8 × 30 s with 30 s intervening cooling sessions on ice. Glass beads, unbroken cells and cell debris was removed by centrifugation at 18 000 *g *for 5 min at 4°C. To the crude extract obtained 3.33 × SDS loading buffer (250 mM Tris-HCl pH 6.8, 40% v/v glycerol, 8% w/v SDS, 2.37 M β-mercaptoethanol, 0.1% w/v Bromophenol Blue) was added for denaturation of the sample (30 min at 20°C). Samples were loaded onto 4-12% gradient SDS gels (NuPAGE^®^Novex^® ^Bis-Tris 4-12% Midi Gels, Invitrogen) for separation of proteins and subsequently transferred to polyvinylidene difluoride (PVDF) membranes (Millipore). Recombinant proteins were visualised by immunodetection (Primary Ab; mouse anti-(H)4, QIAGEN and secondary Ab; polyclonal goat antimouse IgG HRP conjugate, Dako). Densitometric analyses of the western blots, to quantify total protein and degree of degradation for each clone, was performed using the Image J software [47].

#### Large scale expression and membrane preparation

A large scale expression and subsequent membrane preparation was made in order to analyse whether overexpression affected membrane localisation of heterologous protein. A clone with low (HsAQP8:**100**:2) and one with high (HsAQP8:**1000**:1) expression level were compared. The expression was performed in baffled flasks according to the Invitrogen EasySelect™ manual. Briefly, 100 mL BMGY cultures were grown at 28°C and with shaking at 220 rpm over night to OD_600 _= 5. Cells were harvested by centrifugation, resuspended in 500 mL BMMY to OD_600 _= 1 and cultured for 75 h at 28°C with shaking at 200 rpm. Recombinant protein expression was maintained by addition of methanol to a final concentration of 0.5% v/v every 24 h. 3 h after the last methanol addition, cells were harvested by centrifugation and resuspended in 250 mL cold breaking buffer. Cells from 1 L culture (18.6-24.6 g) were broken using a Bead Beater (BioSpec Products) for 10 × 30 s with 30 s intervening cooling sessions. Intact cells and cell debris was removed by centrifugation at 1400 *g *for 2 × 30 min with an intervening resuspension in breaking buffer to ensure efficient recovery of membranes. To collect the total membrane fraction, the 1400 *g *supernatant was centrifuged at 150 000 *g *for 2 h. The membrane pellet was resuspended in Buffer A (20 mM HEPES-NaOH pH 7.8, 50 mM NaCl, 10% v/v glycerol, 2 mM β-mercaptoethanol). All fractions recovered from the membrane preparation procedure were analysed for protein content by immunobloting (as above) after dilution to a normalised volume (corresponding to 270 mL/fraction).

### Determination of copy number by qPCR

#### DNA extraction from P. pastoris

Genomic DNA from the 60 selected clones (HsAQP5:**100**:1-HsAQP5:**1000**:5; HsAQP8:**100**:1-HsAQP8:**1000**:5; SoPIP1;2:**100**:1-SoPIP1;2:**2000**:2 and AtSIP1;1:**100**:1-AtSIP1;1:**1000**:5) and from untransformed X-33 cells was extracted using the Easy-DNA™ Kit (Invitrogen). RNA was removed by incubation with RNase A (Fermentas) at 20°C over night. The DNA was quantified by absorbance measurements at 260 nm.

#### Oligonucleotide primer design

**Figure 6:**
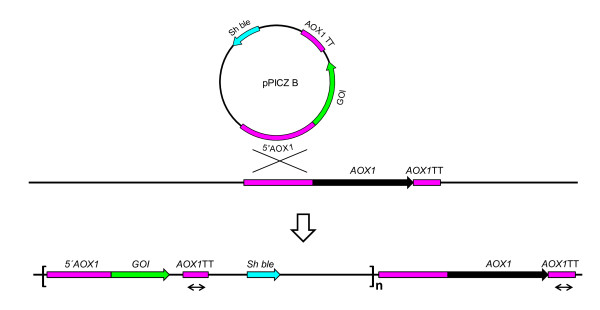
**Integration of the pPICZB vector into the *P. pastoris *genome**. Schematic (adapted from the Invitrogen EasySelect Pichia expression kit manual) of the integration of the pPICZB vector into the *P. pastoris *genome by gene insertion. Double headed arrows indicate the sequence that was amplified using the *PpAOX1 *TT primer pair when quantifying incorporated plasmids by qPCR. To obtain the gene of interest (GOI) copy number (n), the *AOX1 *TT copy number was subtracted by 1 to compensate for the endogenous *AOX1 *TT sequence.

Oligonucleotide primers (Additional file 2, Table S2) were designed using the Primer3 software [48]. For optimal efficiency, primers were designed to generate amplicons of sizes no smaller than 50 bp and no larger than 250 bp. For internal standardisation, a primer pair (*PpAOX2 *prom fw/*PpAOX2 *prom rev) was designed to amplify a stretch of the *AOX2 *promoter sequence, which is present as one copy in the *P. pastoris *genome. To verify number of recombinant gene sequences, another primer pair (*PpAOX1 *TT fw/*PpAOX1 *TT rev) directed towards the 3'TT sequence of the *AOX1 *gene, which is also present in the pPICZ plasmid and is integrated together with the *GOI *(Figure 6), was constructed. The mean efficiency (E) of the two primer pairs was determined according to the serial dilution method [49]. As a template for the standard curve, genomic DNA prepared from X-33 cells was used with concentrations ranging from 1 ng to 100 ng. For each concentration 6 replicates were analyzed. Subsequently to plotting the obtained critical take off (Ct) values against log dilution, primer efficiencies were calculated to 1.95 (AOX2 prom-primers) and 1.94 (AOX1 TT-primers) respectively. The r^2 ^values of the plots were both determined to 0.997. To ensure specificity, melting curve analyses were performed at the end of each qPCR assay. Single peaks corresponding to the PCR products implied that the amplification was specific and that no primer dimers formed during the reactions.

#### qPCR assays

qPCRs were performed using a Corbett Research Rotor Gene thermal cycler (Montreal Biotech). Following pre-incubation at 95°C for 10 min the thermal cycler was programmed to perform 30-40 cycles of: 15 s at 95°C; 30 s at 60°C; 30 s at 72°C. Triplicate or quadruplicate samples of each template were analysed. Individual reactions were carried out in 20 μl volumes containing 10 μL 2 × Maxima™ SYBR^® ^Green qPCR Master Mix (Fermentas), 0.25 μM fw- and rev- primers and 10 ng template DNA.

#### qPCR data analysis

Since the efficiencies of the two primer pairs (*PpAOX2 *prom fw/*PpAOX2 *prom rev and *PpAOX1 *TT fw/*PpAOX1 *TT rev) were nearly identical, they were replaced by a common mean efficiency, thereby simplifying calculations. *AOX1 *TT copy number was determined according to the E^-ΔΔCt^-method [49], described by equation 1 (R_avg _= Average copy number; E = mean primer efficiency (= 1.95); Ct = Critical take off cycle; Sample = Clone compared to reference; Reference = X-33 strain used as reference; A = *AOX1 *TT; B = *AOX2 *promoter). *AOX1 *TT copy numbers plus/minus standard deviation (SD) were calculated according to equation 2, where the standard deviation of ΔCt where calculated according to equation 3 based on the summation rule for variances of independent variables. To obtain the aquaporin copy number, the *AOX1 *TT copy number was subtracted by 1 to compensate for the endogenous *AOX1 *TT sequence.(1)(2)(3)

### Analysis of AtSIP1;1 and HsAQP5 mRNA-levels by qPCR

#### RNA extraction and cDNA synthesis

Total RNA was extracted from induced single copy *Pichia *clones carrying either the AtSIP1;1-construct (AtSIP1;1:**100**:2) or the HsAQP5 construct (HsAQP5:**100**:5) using the PureLink™ RNA Mini Kit (Invitrogen). As a control, RNA was also prepared from uninduced AtSIP1;1:**100**:2 and HsAQP5:**100**:5 cultures. Integrity of the RNA was analysed on a 2100 Bioanalyzer (Agilent Technologies). Samples corresponding to 1.6 μg total RNA were subjected to RNase free DNase (Promega) and cDNA was produced using the RevertAid™ H Minus First Strand cDNA Synthesis Kit (Fermentas) with the oligo dT-primers supplied in the kit.

#### Oligonucleotide primer design

Oligonucleotide primers (Additional file 2, Table S2) were designed using the PrimerPy software (http://code.google.com/p/oligobench/wiki/PrimerPy). Gene specific primers (HsAQP5_fw/HsAQP5_rev and AtSIP1;1_fw/AtSIP1;1_rev) were constructed to amplify the HsAQP5 and AtSIP1;1 cDNA respectively and for internal standardisation primers directed towards the *P. pastoris *actin1 cDNA (PpAct_fw/PpAct_rev) were designed.

#### qPCR assays

qPCR was carried out as described above. 1 μL of the 20 μL cDNA reaction was used as template and samples were run as quadruplicates.

#### qPCR data analysis

Recombinant mRNA levels were calculated as a relative ratio of the actin mRNA levels as described in equation 4 (R_avg _= Relative transcript ratio, E = Mean primer efficiency (1.81 for HsAQP5/Act primers and 1.80 for AtSIP1;1/Act primers), Ct = Critical take off cycle; A = HsAQP5 or AtSIP1;1; B = Act). The standard deviations were calculated as previously described in equation 3.(4)

## Competing interests

The authors declare that they have no competing interests.

## Authors' contributions

KN participated in the design of the study, constructed the *P. pastoris *clones, performed cell culturing and subsequent preparations, carried out immunoblotting assays, qPCR experiments, qPCR data analyses and drafted the manuscript. MA made the vector constructs and participated in the construction of the *P. pastoris *clones and in the immunoblotting assays. JD created the formulas used for qPCR analyses and participated in the drafting of the manuscript. EA contributed to the qPCR experiments. PK participated in the design of the study and in the drafting of the manuscript. UJ conceived the study and participated in the drafting of the manuscript. All authors read and approved of the final manuscript.

## Supplementary Material

Additional file 1 Table S1 Primers used for amplification of cDNA Click here for file

Additional file 2 Table S2 Primers used for qPCR Click here for file
