# Supplementary material for: Increasing gene dosage greatly enhances recombinant expression of aquaporins in Pichia pastoris
Source: BMC Biotechnol. 2011 May 10;11:47. doi: 10.1186/1472-6750-11-47 (PMC3118338; doi:10.1186/1472-6750-11-47)
Supplement: Additional file 1 — Table S1 Primers used for amplification of cDNA [file 1472-6750-11-47-S1.DOCX]

**Table S1** Primers used for amplification of cDNA

| **Primer** | **Primer sequence^1^** | **Construct** | **Source** | **GI^5^** |
| --- | --- | --- | --- | --- |
|  |  |  |  |  |
| *HsAQP5::6×His* fw | 5'AA**GAATTC**AAAATGTCTAAGAAGGAGGTGTGCTCCGTG 3' | HsAQP5::6×His | 5269384 ^2^ | 186910293 |
| *HsAQP5::6×His* rev | 5'AA**GCGGCCGC**GCGGGTGGTCAGCTCCATGG 3' |  |  |  |
| *HsAQP8::6×His* fw | 5'CCC**GAATTC**AAAATGTCTTGTGAGCCTGAATTTGGC 3' | HsAQP8::6×His | 5759105 ^2^ | 45446751 |
| *HsAQP8::6×His* rev | 5'AA**GCGGCCGC**CCGAGCCTTCAGGATGAGGC 3' |  |  |  |
| *AtSIP1;1::6×His* fw | 5'TTT**CTCGAG**AAAATGTCTATGGGTGTGTTGAAGTCGG 3' | AtSIP1;1::6×His | pLS15 ^3^ | 186509744 |
| *AtSIP1;1::6×His* rev | 5'TT**GCGGCCGC**GGCTTTCTTTTGTTTCTTCTTCTG 3' |  |  |  |
| *SoPIP1;2::6×His* fw | 5'GG**GAATTC**AAAATGTCTGAGGGCAAAGATGAAGATGTTAG 3' | SoPIP1;2::6×His | PM28C^4^ | 38532004 |
| SoPIP1;2::6×His rev | 5'CC**GCGGCCGC**CTTGGATTTGAAAGGGATTGC 3' |  |  |  |

^1^ Restriction sites in bold

^2^ IMAGE clone identification numbers

^3^ Unpublished cDNA clone (Dr. Sofia Möller, UJ)

^4^ Previously published cDNA clone [46]

^5^ mRNA gene identifier in GenBank
