# Supplementary material for: Increasing gene dosage greatly enhances recombinant expression of aquaporins in Pichia pastoris
Source: BMC Biotechnol. 2011 May 10;11:47. doi: 10.1186/1472-6750-11-47 (PMC3118338; doi:10.1186/1472-6750-11-47)
Supplement: Additional file 2 — Table S2 Primers used for qPCR [file 1472-6750-11-47-S2.DOCX]

**Table S2** Primers used for qPCR.

| **primer** | **sequence** | **tm (ºC)** | **GC%** | **amplicon**  **length (bp)** | **E**^1^ |
| --- | --- | --- | --- | --- | --- |
| *AOX1* TT fw | 5'-TGGGCACTTACGAGAAGACC-3' | 60.25 | 55 |  |  |
| *AOX1* TT rev | 5'-GCAAATGGCATTCTGACATC-3' | 59.08 | 45 | 65 | 1.94 |
| *AOX2* prom fw | 5'-GACTCTGATGAGGGGCACAT-3' | 60.08 | 55 |  |  |
| *AOX2* prom rev | 5'-TTGGAAACTCCCAACTGTCC-3' | 59.94 | 50 | 139 | 1.95 |
| *AQP5* fw | 5'-CTCAACAACAACACAACGCA-3' | 55.3 | 45 |  |  |
| *AQP5* rev | 5'-CCAGTGAAGTAGATTCCGACAA-3' | 58.4 | 45.5 | 179 | 1.79 |
| *AtSIP1:1* fw | 5'-TTTCAGGCAATCACTTGGG-3' | 54.5 | 47.4 |  |  |
| *AtSIP1:1* rev | 5'-ACACCGGCGACATAAAAGG-3' | 56.7 | 52.6 | 134 | 1.77 |
| *PpAct* fw | 5'-CCAATGAACCCAAAGTCCAA-3' | 55.3 | 45 |  |  |
| *PpAct* rev | 5'-CCGTCACCAGAGTCCAAAAC-3' | 59.4 | 55 | 149 | 1.83 |
|  |  |  |  |  |  |
| ^1^ Primer efficiency as determined by the serial dilution method [49] | | | | | |
